# Exploring Wearable Device Use Among Dementia Caregivers: Benefits, Challenges, and Improvements

**DOI:** 10.1093/geroni/igaf122.3439

**Published:** 2025-12-31

**Authors:** Jung-Ah Lee, Hyun Jung Kim, Kyeung Mi Oh

**Affiliations:** University of California, Irvine, Irvine, California, United States; Seattle University, Seattle, Washington, United States; George Mason University, Fairfax, Virginia, United States

## Abstract

Caregivers of persons with dementia experience significant challenges and stress due to round-the-clock responsibilities. Wearable devices (WD), such as smartwatches and smart rings, can provide real-time health data, enhancing caregivers’ self-care. This study explored caregivers’ experiences using WD as part of a three-month in-home educational intervention, which included stress management strategies. Participants (N = 101) were recruited through community outreach and completed exit interviews. The sample included 14 non-Hispanic White, 13 Hispanic, 35 Korean, and 39 Vietnamese caregivers. Qualitative analysis identified four key themes: enhancing usability, encouraging data engagement, improving wearability and convenience, and expanding health-tracking features. Most caregivers found WD beneficial for increasing health awareness, motivation, and self-monitoring. However, they highlighted the need for additional education on device functions, app navigation, and data interpretation. Non-English-speaking caregivers emphasized the importance of language accessibility. Many preferred larger screens for better readability and reminders to improve adherence. Some struggled with discomfort, forgetting to wear or charge devices, and limited battery life. Caregivers expressed interest in additional health-tracking features, including blood pressure, blood sugar, body fat, and muscle mass monitoring. Addressing these challenges could enhance adherence, particularly among older caregivers. Participants recommended tailored education for caregivers with varying levels of digital literacy, alternative charging methods, and visible battery indicators to improve usability. Findings highlight the need for comprehensive education, frequent reminders, and improved usability to maximize WD benefits. By addressing these factors, WD can better support caregivers in managing their well-being and sustaining their caregiving roles with greater resilience.

